# Cartas al editor

**DOI:** 10.7705/biomedica.7247

**Published:** 2023-12-01

Santiago de Cali, 15 de octubre de 2023

Señores

Comité Editorial

Bogotá

Estimados señores:

Nos permitimos enviarles un comentario sobre el artículo “Brote de *tinea capitis* tricofítica en un grupo de niños escolares en un área rural del departamento del Cauca, Colombia” (Biomédica. 2023;43(Sp.1):57-68. https://www.doi.org/10.7705/biomedica.6793) para la sección de cartas al editor.

Atentamente,

José Ivo Montaño Caicedo

Profesor Asistente, Universidad del Valle

Carlos Andrés Pineda Cañar

Profesor Titular, Universidad del Valle

**Comentario**

El reporte de caso publicado por González y colaboradores ^1^ ilustra la actuación de las autoridades de salud pública y su articulación con una institución educativa en el abordaje epidemiológico y diagnóstico de un brote de *tinea capitis* en una comunidad. La identificación de las condiciones que favorecen la transmisión de las micosis cutáneas y de su agente causal permite orientar las medidas preventivas y terapéuticas, para garantizar que las familias cuenten con opciones para cuidar su salud y recibir el tratamiento adecuado. En estos casos, la griseofulvina, la terbinafina o el itraconazol por vía oral han mostrado las tasas más altas de curación ^2^^,^^3^.

*Tinea capitis* es una enfermedad de interés en la práctica de la medicina familiar dada su alta prevalencia, disponibilidad de herramientas diagnósticas, tratamientos efectivos y necesidad de abordaje familiar y comunitario, razón por la cual revisamos con detenimiento el reporte de González *et al*. ^(^^1^. Llamó nuestra atención la afirmación “sólo tres niños (9,4 %) recibieron tratamiento completo”, pues se esperaría que la cobertura fuese del 100 %. Sin embargo, esta cifra podría ser un indicador de la escasa efectividad de las acciones reportadas, consistentes en remitir información a tres entidades: secretaría de salud, entidades promotoras de salud y hospital local.

No obstante, la baja cobertura de tratamiento está influenciada por la falta de accesibilidad a los servicios de salud, como lo menciona el mismo reporte ^1^, que evidencia fallas en los cuatro atributos distintivos de la atención primaria en salud ^4^: primer contacto, seguimiento longitudinalidad, integralidad y coordinación. En el ámbito del primer contacto, no se reporta un proveedor de servicios sanitarios a quien los afectados puedan acudir; en la longitudinalidad se incluye la falta de seguimiento a los afectados, mientras que en la integralidad está consignado el hecho de que no se suministra tratamiento para la enfermedad a todas las personas afectadas; y en cuanto a la coordinación, los autores no reportan articulación con los servicios sanitarios para apoyar el tratamiento, el seguimiento y la prevención de otros casos y recurrencias.

También podría soslayarse la falta de tratamiento por el efecto de las políticas públicas de salud en la accesibilidad a los servicios sanitarios, ya que promueven gradientes de desigualdad. Por ejemplo, las Rutas Integrales de Atención en Salud son elaboradas para diversas entidades desde el paradigma de atención primaria en salud selectiva ^5^. Por lo tanto, una enfermedad con una ruta integral de atención podría tener mayor acceso a servicios de salud que una sin una ruta definida, como la *tinea capitis.* La función de articulación que incluye contratación, referencia, contrarreferencia, atención especializada y redes integrales, entre otras, asignada exclusivamente a las entidades promotoras de salud, determina que las personas con más recursos para el agenciamiento podrían tener mayor acceso que las personas con menos recursos. La promoción de la salud reducida a una lista de actividades asistenciales que instrumentaliza el autocuidado, la educación y la participación como medios para lograr su cobertura poblacional, podría explicar que personas con preferencias afines a este enfoque gocen de mayor accesibilidad que aquellas con preferencias diferentes.

Por otro lado, la exclusión de la griseofulvina del plan de beneficios de Colombia, aunque haga parte de la lista de medicamentos esenciales ^6^, y esté en la primera línea de tratamiento, reduce las alternativas terapéuticas de las personas con *tinea capitis*.

Además, podrían enunciarse factores familiares que afectan el cumplimiento del tratamiento como el nivel cooperación entre el niño y sus padres, la falta de síntomas en otros miembros de la familia, el temor a la estigmatización o la duración del tratamiento ^7^.

Dado que el propósito de nuestra lectura del reporte se enfocó en indagar sobre el comportamiento de la tinea capitis en nuestro entorno para ajustar nuestra práctica clínica, consultamos si la Organización Mundial de la Salud (OMS) tenía alguna recomendación respecto al tratamiento de los pacientes durante el abordaje de brotes por enfermedades infecciosas, lo cual en efecto está contemplado en los capítulos siete y ocho de la “Guía para el manejo de asuntos éticos en brotes de enfermedades infecciosas” ^8^. Con esta información concluimos que la OMS recomienda incluir el tratamiento como parte del abordaje de brotes por enfermedades infecciosas. Por lo cual, sugerimos respetuosamente hacer lo que esté al alcance de cada lector, para que, en próximos abordajes de brotes por *tinea capitis* en Colombia, aunque no se publiquen, se pueda verificar la observación de las recomendaciones de la OMS ^8^.
