# Supplementary material for: Cartas al editor
Source: Biomedica. 2023 Dec 1;43(4):534–7. [Article in Spanish] doi: 10.7705/biomedica.7247 (PMC10793873; doi:10.7705/biomedica.7247)
Supplement: Related texts

# Reply: Cartas al editor

Popayán, 15 de noviembre de 2023

Señores

Comité editorial

Revista Biomedica

Estimados señores:

Atento saludo, me permito remitir la respuesta enviada por uno de los autores del manuscrito “Brote de *tinea capitis* tricofítica en un grupo de niños escolares en un área rural del departamento del Cauca, Colombia”. Biomédica. 2023;43(Sp.1):57-68. https://www.doi.org/10.7705/biomedica.6793

Nos parece muy importante recibir comentarios sobre el artículo que muestra una realidad sobre la desarticulación de los actores del sistema en contextos de pobreza, vulnerabilidad, carencia de saneamiento básico, violencia -dada la presencia de diversos grupos armados- y escaso desarrollo institucional del sector sanitario.

Es difícil llevar a cabo planes de contingencia en casos fortuitos de eventos epidemiológicos, en particular, cuando los municipios son categoría seis y no cuentan con personal para la atención de brotes y otras situaciones de salud pública que lo requieran. De manera que los hospitales locales cobran gran relevancia porque deben ejecutar acciones ya sea que existan o no contrataciones específicas para casos como la serie presentada de *tinea capitis*.

A la fecha, no existe en Colombia una guía específica de atención para situaciones como la que describe el artículo, y mucho menos están establecidos los lineamientos sobre el tipo y la dosis de medicamentos que se deben aplicar. Aunque el hospital local participó activamente en la prescripción de los medicamentos utilizados, el acceso oportuno a ellos es difícil, porque su distribución depende no solo de las entidades prestadoras de servicios de salud, sino de otros intermediarios, lo cual implica demoras en su distribución y, por ende, en su administración. No obstante, el hospital cuenta con un programa para atender casos de micosis así como de otras enfermedades desatendidas, algunas de las cuales se han priorizado en la política pública del país y son objeto de estrategias específicas que buscan eliminar su transmisión. Tal es el caso de la oncocercosis, el tracoma y otras. Esta serie de casos de *tinea capitis* indudablemente hace que se preste mayor atención a dichos eventos.

Cabe mencionar que la Universidad del Cauca no contó con recursos para ninguna de las actividades realizadas, a pesar de aplicar a varias fuentes de financiación. Por esta razón, es importante que existan recursos destinados exclusivamente a fortalecer la investigación operativa, la articulación institucional estatal y la participación comunitaria, para abordar la temática de las micosis como problema de salud pública. Es clara la necesidad de programas específicos para la atención individual y colectiva de las micosis y su enlace con otros programas como los de salud escolar integral.

Giovanny Apráez

Ph.D. en Salud Pública
